# GALAD outperforms aMAP and ALBI for predicting HCC in patients with compensated advanced chronic liver disease: A 12-year prospective study

**DOI:** 10.1097/HC9.0000000000000262

**Published:** 2023-09-15

**Authors:** Erica Villa, Rossella Donghia, Valentina Baldaccini, Calogero C. Tedesco, Endrit Shahini, Raffaele Cozzolongo, Sara Ascari, Pasqua Letizia Pesole, Sergio Coletta, Rosina Maria Critelli, Simone Lasagni, Filippo Schepis, Filippo Semellini, Gianluigi Giannelli

**Affiliations:** 1 Gastroenterology Unit, CHIMOMO Department, University of Modena & Reggio Emilia, Italy; 2 National Institute of Gastroenterology, IRCCS “Saverio de Bellis”, Castellana Grotte, Italy; 3 Clinical and Experimental Medicine PhD Program, University of Modena and Reggio Emilia, Modena, Italy

## Abstract

**Background and Aims:**

Surveillance programs are strongly recommended in patients with liver cirrhosis for early detection of HCC development. Six-monthly ultrasound sonography is the most reliable and commonly used technique, especially when associated with serum determination of α-fetoprotein, but different score systems have been proposed to overcome the unsatisfactory diagnostic accuracy of α-fetoprotein. The aim of this 12-year prospective study is to compare the gender, age, AFP-L3, AFP, des-gamma-carboxy prothrombin (GALAD) versus age, gender, bilirubin, albumin, and platelets and albumin-bilirubin scores in predicting HCC onset.

**Approach and Results:**

A cohort of 545 consecutive patients with compensated advanced chronic liver disease without suspected focal lesions was followed up every 6 months by liver imaging and α-fetoprotein to detect HCC occurrence. Harrell’s C-index for censored data was employed to evaluate the performance of any parameters or scores helping to predict HCC development. ROC curve analysis showed that the GALAD score was more accurate in evaluating HCC development than albumin-bilirubin and age, gender, bilirubin, albumin, and platelets. The AUC ranged from 0.7268 to 0.6851 at 5 and 10 years, both in the total cohort and in the sub-cohorts (viral hepatitis, NASH, and alcohol). The HCC Risk model was constructed using univariate and multivariate Cox proportional hazard regression analysis, showing a strong association of GALAD with HR > 1, *p* < 0.05, in the total and sub-cohorts, and a better risk prediction in the alcohol cohort, both alone and standardized with other blood parameters.

**Conclusions:**

GALAD is the most reliable and accurate score system to detect HCC risk of development in patients with compensated advanced chronic liver disease.

## Graphical abstract

**Figure:**
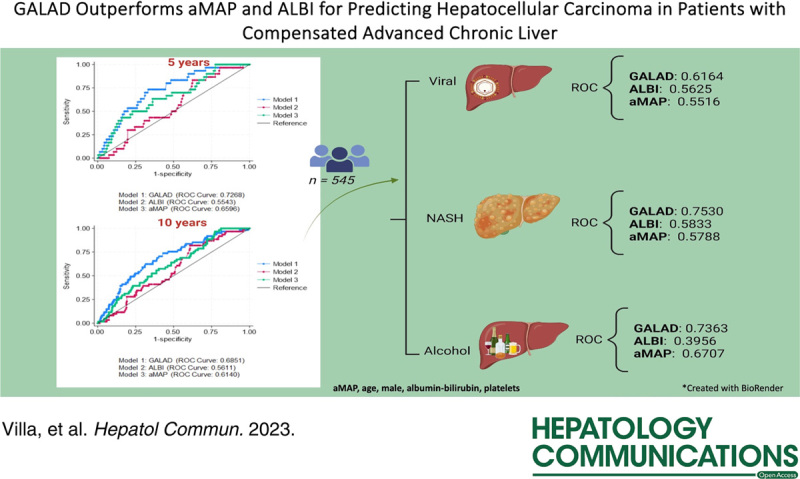

## INTRODUCTION

Liver cancer is the sixth most common cancer worldwide but also the second leading cause of cancer death reported in GLOBOCAN 2020 cancer estimates:^1^ new cases and deaths are expected to rise more than 50% by 2040.^2^ Incidence and mortality vary across countries, with the highest rates in Eastern Asia, Northern Africa, and South-Eastern Asia, and by gender, the male/female ratio being around 2:1. Globally, HCC accounts for 80% of all cases of primary liver cancer, followed by intrahepatic cholangiocarcinoma (ICC) at 15%, and other subtypes accounting for the remaining 5%.^3^

Prognosis and quality of life are directly dependent on the stage of HCC and treatments available at the time of diagnosis.^4^ About 60% of the patients receiving the most effective therapeutic options (liver transplantation, surgery, and radiofrequency) at the time of diagnosis have a 5-year survival rate versus only 1–2 years in patients with more advanced disease.^5,6^

HCC occurs in more than 90% of the patients with underlying chronic liver disease (CLD), cirrhosis being the most important risk factor, independent of etiology.^6^ As a result, all scientific societies strongly advocate early HCC detection programs in high-risk patients, such as those with liver cirrhosis. The American Association for the Study of Liver Diseases AASLD recommends surveillance when the risk of HCC is at least 1.5%/year and the incidence is greater than 0.2%/year. The strongest evidence for HCC surveillance derives from a 20-year randomized controlled trial in China in which a total of 18,816 patients with CLD undergoing 6-monthly screening showed a 37% reduced HCC mortality.^7^

The increase in life expectancy among patients with cirrhosis treated as a result of early HCC detection during surveillance programs exceeds 3 years.^8^

While the overall effectiveness of 6-monthly surveillance programs is currently unquestionable, how to improve it is still a matter of debate. Abdominal ultrasonography (US) is the most commonly used technique owing to its wide availability and low cost.^8^ However, US detection of HCC is limited by several factors, such as abnormal liver texture, obesity, and the quality of US images. In terms of biomarkers, α-fetoprotein (AFP) is most commonly used for HCC surveillance. A meta-analysis performed in 2018 in the United States showed that AFP used alone improves detection accuracy in clinical practice for HCC at any stage and also at an early stage, albeit with a moderate decrease in specificity.^9^ AFP received a ‘conditional’ recommendation for use in conjunction with a 6-monthly US according to AASLD guidelines, but little is yet known about the performance of this combination or US plus other biomarkers.

Therefore, improved biomarker performance remains an unmet need, although recently, various predictive scores have been proposed and investigated.

A score built from a large international database based on albumin-bilirubin (ALBI) tests was proposed in 2015 as an alternative to the Child-Pugh classification, but also as a predictor of the onset of HCC, then confirmed in a subsequent study.^10,11^ An international collaboration including 11 prospective observational cohorts or randomized controlled trials developed a new HCC risk score based on age, gender, bilirubin, albumin, and platelets (aMAP), which was validated as a reliable and accurate score for guided-surveillance strategies.^12^ Gender, age, AFP-L3, AFP, des-gamma-carboxy prothrombin (GALAD), the most recently proposed score system, has demonstrated high sensitivity in detecting HCC in patients with cirrhosis.^13^

The purpose of this study is to compare the most validated scores for the prediction of HCC, aMAP, and ALBI versus GALAD in a prospective cohort of patients with compensated advanced chronic liver disease over a 12-year follow-up.

## METHODS

### Study population

We studied a cohort of 545 consecutive patients with compensated advanced chronic liver disease, recruited between 2010 and 2018, in the Gastroenterology Department of the University of Modena and Reggio Emilia. All patients were followed up, reaching a median of 3.60 years at 5 years, and 5.58 years at 10 years. At the time of enrolment, all the patients had Child-Pugh A or B cirrhosis with no evidence of any HCC lesion, as determined by a CT scan and/or MRI. Exclusion criteria included the presence of suspicious lesions at US/CT or MRI, diagnosis of HCC during the first 6 months of follow-up, and any past decompensation event, such as ascites, encephalopathy, or variceal bleeding.

For 12 years, patients underwent an HCC surveillance program based on 6-monthly liver imaging and AFP until incident HCC, liver transplantation, death, or loss to follow-up.

The study protocol (IRB10/08_CE_UniRer; IRB239/12_CE_UniRer2; ClinicalTrials ID: NCT01657695; NCT03083002) was approved by the Ethics Committee of Azienda Ospedaliero-Universitaria, Modena, and each patient provided written informed consent. The study met the principles of the Helsinki Declaration and adhered to the “Standards for Reporting Diagnostic Accuracy Studies” guidelines (http://www.stard-statement.org/) and the “Strengthening the Reporting of Observational Studies in Epidemiology” guidelines.

### Score calculation

The immunoanalyzer TASWakoTM i30, Micro Total Analysis System (FUJIFILM Wako Pure Chemical Corporation, Chuo-Ku Osaka, Japan) was used to determine the serum levels of AFP, AFP-L3, and des-gamma-carboxy prothrombin (DCP). This automated system quantitatively measures the biomarker concentrations by microfluidic electrophoretic separation. The reportable range of AFP concentration is 0.3–1000 ng/ml. The reportable range of AFP-L3% is 0.5–99.5%. AFP-L3% is calculated as follows: AFP-L3% = AFP-L3 concentration/(AFP-L1 concentration + AFP-L3 concentration) × 100. The limit of detection for AFP-L1 and AFP-L3 was found to be 0.030 and 0.028 ng/ml, respectively. The reportable range of DCP concentration is 0.1–950 ng/ml. Expected values are less than 7.5 ng/ml. The limit of detection for DCP was found to be 0.042 ng/ml.

The combination of these 3 biomarkers plus age and gender provides the GALAD score, calculated as − 10.08 + 1.67 × Gender (1 for males, 0 for females) + 0.09 × Age + 0.04 × AFP-L3% + 2.34 × log_10_AFP + 1.33 × log_10_DCP. The aMAP score includes age, gender, bilirubin, albumin, and platelets using the following equation: ({0.06 × Age + 0.89 × Gender (1 for males, 0 for females) + 0.48 × (log_10_ (Bilirubin×0.66)−0.085× Albumin) −0.01 × Platelets} + 7.4 )/14.77×100.^14^

The ALBI score is computed as follows: ALBI score = log_10_(Bilirubin × 0.66)−0.085 × Albumin. The APRI score was calculated using 40 IU/L as the value for the aspartate aminotransferase (AST) upper limit by the formula: APRI score = ((AST/AST upper limit of normal value/PLT)) × 100.

To verify whether the addition of albumin, platelets, and bilirubin (ie, the clinical parameters present in the ALBI and aMAP score) improved GALAD performance, we created 2 additional scores (GALAD/Alb/Bil [z-score] and GALAD/Alb/Bil/Plt [z-score] in which the above-mentioned parameters were added.

### Statistical analysis

Patients’ characteristics are reported as the median and minimum and maximum value for continuous variables and frequency and percentage (%) for categorical variables. To test the association between the independent groups (HCC vs. non-HCC), chi-square test was used for categorical variables, where necessary, and the Wilcoxon rank Mann-Whitney test for continuous variables.

The associations of variables or scores with HCC risk were evaluated by Cox proportional hazards regression model. Harrell C-index (C-Index)for censored data was employed to evaluate the predictive performance of any parameters or scores helping to predict HCC development.

Analyses were made of the total cohort, viral hepatitis, NASH, and alcoholism sub-cohorts. A competing risk analysis was also performed and reporting sub-distribution HR (SHR) with 95% CI using the Fine and Gray method. To test the null hypothesis of non-association, the 2-tailed probability level was set at 0.05. The analyses were conducted using StataCorp. 2023. Stata Statistical Software: Release 18. College Station, TX: StataCorp LLC.

## RESULTS

In our prospective study, we observed overall 78 HCC per 3042 person-years, resulting in a 2.4% yearly incidence over a total median follow-up of 5.58 years. The baseline characteristics of the sample, subdivided into patients who later developed HCC and those who did not, are reported in Table 1. The HCC group was slightly but not statistically significantly older, and there was a more significant prevalence of males with HCC (*p* = 0.02). HCC patients were slightly overweight, and the higher rate of diabetes than liver cirrhosis nearly reached significance.

**TABLE 1:** Epidemiological and clinical characteristics of patients at baseline, divided according to the later development of HCC during follow-up or no development

Parameters^a^	Total Cohort (n = 545)	No HCC (n = 467)	HCC (n = *78*)	*p* ^b^
Gender (M) (%)	360 (66.06)	299 (64.16)	61 (77.22)	0.02^c^
Age (y)	59.00 (17.00–85.00)	58.00 (17.00–83.00)	61.00 (39.00–85.00)	0.13
BMI (Kg/m^2^)	26.25 (14.98–46.94)	26.26 (14.98–46.94)	25.87 (19.26–39.30)	0.77
Diabetes (Yes) (%)	135 (25.05)	113 (24.57)	22 (27.85)	0.53^c^
Etiology (%)	—	—	—	0.001^c^
ALD	124 (26.74)	114 (29.61)	10 (12.00)	—
NASH	100 (21.52)	87 (22.34)	13 (17.33)	—
HCV	192 (41.74)	153 (39.74)	39 (52.00)	—
HBV	46 (10.00)	32 (8.31)	14 (18.67)	—
Chemistry				
Blood Sugar (mg/dL)	97.00 (66.00–387.00)	97.00 (66.00–228.00)	97.00 (74.00–387.00)	0.79
Albumin (g/dL)	3.82 (0.00–5.00)	3.84 (0.00–5.00)	3.80 (2.40–4.90)	0.08
Bilirubin (mg/dL)	0.96 (0.20–32.30)	0.94 (0.20–32.30)	0.99 (0.30–6.70)	0.22
Creatinine (mg/dL)	0.79 (0.41–7.91)	0.80 (0.41–7.91)	0.78 (0.52–4.26)	0.40
Platelets (10^3^/µL)	109.00 (18.00–788.00)	111.00 (18.00–788.00)	92.00 (33.00–340.00)	0.02
Iron (mcg/dL)	85.00 (13.00–622.00)	83.50 (13.00–622.00)	94.50 (15.00–288.00)	0.18
INR	1.20 (0.10–3.02)	1.19 (0.10–3.02)	1.21 (0.89–2.97)	0.32
AST (IU/L)	29.00 (6.00–545.00)	29.00 (6.00–454.00)	26.50 (7.00–182.00)	0.73
ALT (IU/L)	32.00 (5.00–821.00)	31.50 (5.00–821.00)	33.00 (9.00–306.00)	0.41
GGT (IU/L)	52.00 (7.00–726.00)	53.50 (7.00–726.00)	51.00 (17.00–180.00)	0.87
AFP (ng/mL)	4.00 (0.20–680.30)	3.80 (0.20–416.00)	7.65 (0.90–680.30)	< 0.0001
AFP-L3 (%)	0.20 (0.20–70.50)	0.20 (0.20–70.50)	4.95 (0.20–12.620)	0.003
DCP (ng/mL)	0.46 (0.01–950.00)	0.45 (0.01–950.00)	0.49 (0.12–767.43)	0.44
Scores				
MELD	90.00 (1.00–29.31)	9.00 (1.00–29.31)	9.00 (5.00–21.00)	0.88
Child-Pugh	—	—	—	0.40^c^
A	383 (72.54)	329 (72.47)	54 (72.97)	—
B	104 (19.70)	87 (19.16)	17 (22.97)	—
C	41 (7.77)	38 (8.37)	3 (4.05)	—
ALBI	−2.47 (−3.52–0.71)	−2.48 (−3.52–0.71)	−2.42 (−3.32–0.80)	0.06
aMAP	63.18 (16.62–83.47)	62.62 (16.62–83.47)	65.61 (54.92–78.71)	0.001
GALAD	−2.29 (−7.83–6.58)	−2.46 (−7.83–6.58)	−1.39 (−7.14–2.71)	< 0.0001

a As median and range (minimum and maximum value) for continuous and percentage (%) for categorical variables.

b Wilcoxon rank-sum test (Mann-Whitney).

c Chi-Square or Fisher’s test, where necessary.

Abbreviations: AFP, alpha fetoprotein; AFP, des-gamma-carboxy prothrombin; ALBI, albumin-bilirubin; ALD, alcoholic liver disease; ALT, alanine aminotransferase; aMAP, age-male-albumin-bilirubin-platelets score; AST, aspartate aminotransferase; BMI, body mass index; DCP, des-gamma-carboxy prothrombin; GALAD, gender, age, AFP-L3; GGT, gamma-glutamil-transferase; INR, international normalized ratio; MELD, Model for End-Stage Liver Disease.

A strong association, with clearly different proportions between the 2 groups, was demonstrated for the presence of different etiology (*p* = 0.001).

Platelet levels were significantly lower in the HCC than in non-HCC groups (*p* = 0.02). No differences were observed for the other blood parameters, albumin, bilirubin, creatinine, AST, alanine aminotransferase, iron, International Normalized Ratio, and gamma glutamyl transferase. Variables included in the GALAD score, AFP, and AFP-L3 were strongly and statistically significantly (*p* < 0.0001, and *p* = 0.003) increased in HCC patients. The ALBI, aMAP, and GALAD scores confirmed the notable differences between the independent groups analyzed (*p* = 0.06, *p* = 0.001, and *p* < 0.0001, respectively). Univariate Cox regression models to test the associations for HCC at 5, 7, and 10 years of follow-up and the respective performance (C-Index) are reported in Table 2. Demographic and blood parameters, such as age, gender, viral etiology, AFP, and DCP, showed positive associations with the highest risk for HCC at 5 years, whereas higher albumin levels had an inverse relationship with HCC prediction. The same trend emerged at 7 and 10 years. Child-Pugh, ALBI, aMAP, GALAD scores, and standardized variables were strongly associated with the HCC risk, *p* < 0.05.

**TABLE 2:** Cox regression model on single parameters for HCC prediction at 5, 7, and 10 years

	5 y				7 y				10 y			
Parameters	HR	*p*	95% (CI)	C-Index	HR	*p*	95% (CI)	C-Index	HR	*p*	95% (CI)	C-Index
Age	1.04	**0.01**	1.01–1.07	0.58	1.03	**0.009**	1.01–1.06	0.57	1.03	**0.006**	1.01–1.05	0.58
Gender (M)	2.41	**0.03**	1.06–5.46	0.58	2.00	**0.03**	1.08–3.69	0.58	1.88	**0.02**	1.10–3.23	0.57
BMI	1.02	0.57	0.95–1.10	0.55	1.02	0.44	0.96–1.09	0.54	1.03	0.27	0.98–1.08	0.55
Diabetes	1.39	0.36	0.69–2.79	0.54	1.51	0.13	0.88–2.60	0.56	1.37	0.21	0.83–2.25	0.55
Viral Etiology	2.43	**0.02**	1.18–5.01	0.61	2.19	**0.005**	1.26–3.80	0.60	1.96	**0.007**	1.20–3.18	0.59
Albumin	0.47	**0.004**	0.28–0.79	0.62	0.50	**0.001**	0.34–0.75	0.63	0.54	**0.001**	0.37–0.78	0.63
Bilirubin	1.03	0.79	0.83–1.28	0.57	1.07	0.19	0.97–1.19	0.59	1.05	0.33	0.95–1.18	0.57
AST	0.99	0.28	0.98–1.00	0.50	1.00	0.70	0.99–1.00	0.59	1.00	0.97	0.99–1.00	0.58
ALT	0.99	0.62	0.99–1.00	0.41	0.99	0.91	0.99–1.00	0.46	0.99	0.49	0.99–1.00	0.50
AFP	1.00	**0.05**	1.00–1.01	0.62	1.00	0.09	0.99–1.01	0.57	1.00	< **0.001**	1.00–1.01	0.58
AFP-L3	1.03	0.08	0.99–1.06	0.60	1.02	0.28	0.99–1.05	0.53	1.01	0.35	0.98–1.04	0.52
DCP	1.00	**0.03**	1.00–1.01	0.69	1.00	0.15	0.99–1.00	0.67	1.00	0.18	0.99–1.00	0.65
*Scores*												
MELD	1.05	0.18	0.98–1.14	0.58	1.04	0.18	0.98–1.11	0.59	1.03	0.30	0.97–1.10	0.57
Child-Pugh	1.29	0.43	0.68–2.42	0.51	1.73	**0.01**	1.13–2.64	0.59	1.55	**0.03**	1.04–2.31	0.58
ALBI Score	1.97	**0.009**	1.19–3.27	0.62	1.96	**0.001**	1.32–2.92	0.63	1.81	**0.001**	1.26–2.60	0.62
aMAP	1.13	< **0.001**	1.07–1.20	0.62	1.08	< **0.001**	1.04–1.13	0.60	1.09	< **0.001**	1.05–1.13	0.61
GALAD	1.45	< **0.001**	1.23–1.71	0.72	1.39	< **0.001**	1.23–1.57	0.70	1.36	< **0.001**	1.21–1.52	0.70
GALAD/Alb/Bil (z-score)	1.30	**0.002**	1.10–1.54	0.69	1.28	< **0.001**	1.12–1.45	0.67	1.24	< **0.001**	1.11–1.40	0.66
GALAD/Alb/Bil/Plt (z-score)	1.21	**0.02**	1.03–1.42	0.67	1.22	**0.002**	1.08–1.38	0.66	1.17	**0.006**	1.05–1.31	0.65

Abbreviations: C-Index, Harrell C-index.

All the individual statistically significant variables are included in the model shown in Table 3, obtaining a better prediction performance (C-Index>0.70) at each time point. Age, gender, and albumin kept a strong association with HCC at 5, 7, and 10 years, respectively, with the same trend as in univariate analysis (Table 2). In the multivariate model against the other scores, the GALAD score was reconfirmed as a better predictor at each time point (*p* = 0.003, *p* < 0.0001, and *p* = 0.001, respectively) while the other scores were completely outperformed, with only ALBI showing a borderline significance at 10 years. The GALAD model showed superior performance in the detection of HCC at 5, 7, and 10 years compared to the other models (AUC = 0.7268, AUC = 0.7087, and AUC = 0.6851, *p* = 0.002 at 5 and 7 years, and *p* = 0.04 at 10 years). Standardized GALAD showed better performance than GALAD/Alb/Bil and GALAD/Alb/Bil/Plt. Univariate Cox models were also built for the other sub-cohorts. The Viral hepatitis cohort (Supplemental Table 1, http://links.lww.com/HC9/A511) showed an inverse association for albumin at all time points (HR = 0.37, HR = 0.44, and HR = 0.48, respectively, *p* < 0.05) in predicting HCC risk, and AFP showed a significant risk only at the last follow-up (*p* < 0.001). The Child-Pugh, ALBI, aMAP, and GALAD scores revealed statistically significant risk factors, but only GALAD preserved statistical significance. Significant variables at univariate analysis were included together in the model as shown in Supplemental Table 2, http://links.lww.com/HC9/A511, demonstrating the good performance of GALAD from 5 to 10 years. Surprisingly, in the NASH cohort (Supplemental Table 3, http://links.lww.com/HC9/A511), only albumin was associated with HCC (*p* = 0.05) at 7 years as a protective factor. ALBI and standardized GALAD were associated only at 7 years (*p* = 0.05, and *p* = 0.04, respectively) and 10 years.

**TABLE 3:** Multiple Cox regression model for HCC prediction at 5, 7, and 10 years

	5 y				7 y				10 y			
Parameters	HR	*p*	95% (C.I.)	C-Index	HR	*p*	95% (C.I.)	C-Index	HR	*p*	95% (C.I.)	C-Index
Age	1.07	0.002	1.02–1.11	0.75	1.04	0.004	1.01–1.06	0.71	1.04	0.004	1.01–1.06	0.71
Gender (M)	4.19	0.009	1.43–12.30	—	2.43	0.01	1.23–4.77	—	1.98	0.03	1.07–3.68	—
Viral Etiology	1.86	0.20	0.72–4.81	—	2.05	0.02	1.11–3.77	—	1.68	0.09	0.92–3.06	—
Albumin	0.34	0.009	0.15–0.77	—	0.49	< 0.001	0.33–0.72	—	0.44	0.001	0.26–0.73	—
AFP	1.00	0.62	0.99–1.01	—	—	—	—	—	1.00	0.001	1.00–1.01	—
DCP	1.00	0.61	0.99–1.00	—	—	—	—	—	—	—	—	—
*Scores*												
Child-Pugh	—	—	—	0.71	0.89	0.74	0.43–1.82	0.71	0.74	0.38	0.38–1.45	0.70
ALBI Score	1.50	0.29	0.70–3.21	—	1.95	0.07	0.95–4.03	—	1.92	0.05	1.00–3.68	—
aMAP	1.06	0.10	0.99–1.15	—	1.01	0.60	0.96–1.07	—	1.03	0.24	0.98–1.08	—
GALAD	1.33	0.003	1.10–1.60	—	1.31	< 0.001	1.13–1.51	—	1.26	0.001	1.10–1.44	—

Abbreviations: C-Index, Harrell C-index.

Age was associated with HCC risk in the alcohol sub-cohort, with significant *p* values (*p* = 0.02, *p* = 0.01, and *p* = 0.009) (Supplemental Table 4, http://links.lww.com/HC9/A511) at each time point, and the blood parameters AST and alanine aminotransferase were risk factors, *p* < 0.05. GALAD alone, and also standardized with other variables, was a risk factor with a high HR and statistically significant *p*-value (*p* = 0.01, *p* = 0.001, and *p* = 0.001 at each time point). All parameters included together in the model (Supplemental Table 5, http://links.lww.com/HC9/A511) showed a significant association only for age at the last 2 time points (*p* = 0.01, and *p* = 0.004, respectively), while among blood parameters, only AST was a risk factor for HCC at 10 years. GALAD remained the optimal score to predict HCC also in this sub-cohort, but only at 10 years.

Sub-cohort analysis based on different etiologies was performed to assess the accuracy of the investigated scores. Table 4 shows the AUC of GALAD, ALBI, and aMAP at 5, 7, and 10 years, respectively, for each sub-cohort. No differences were found between models in the viral hepatitis cohort. In the NASH sub-cohort, GALAD performance was good, especially at 7 and 10 years, while the other 2 scores, and above all ALBI, had a worse performance. At 10 years, GALAD had a better performance (*p* = 0.02) than ALBI, and aMAP. In the alcohol-associated sub-cohort, the GALAD outperformed the other 2 scores at each time point (*p* = 0.0003, *p* = 0.008, and *p* = 0.002, respectively), and AUC 0.8387, 0.7633, and 0.7363, respectively.

**TABLE 4:** Area under the receiver operating characteristic (ROC) curves of scores at 5, 7, and 10 years

Parameters	AUC	se (AUC)	95% (CI)	*p* ^a^
Viral cohort				
5 y				
GALAD	0.6571	0.06	0.54–0.77	0.64
ALBI	0.5765	0.06	0.46–0.69	—
aMAP	0.6190	0.07	0.49–0.75	—
7 y				
GALAD	0.6291	0.05	0.53–0.72	0.26
ALBI	0.5525	0.05	0.45–0.66	—
aMAP	0.5295	0.06	0.42–0.64	—
10 y				
GALAD	0.6164	0.05	0.52–0.71	0.52
ALBI	0.5625	0.05	0.47–0.66	—
aMAP	0.5516	0.05	0.45–0.65	—
NASH Cohort				
5 y				
GALAD	0.6810	0.18	0.33–0.99	0.62
ALBI	0.5632	0.11	0.34–0.78	—
aMAP	0.6724	0.23	0.23–0.99	—
7 y				
GALAD	0.7610	0.14	0.47–0.99	0.23
ALBI	0.6360	0.11	0.42–0.85	—
aMAP	0.6754	0.16	0.36–0.99	—
10 y				
GALAD	0.7530	0.10	0.56–0.95	0.02
ALBI	0.5833	0.09	0.41–0.75	—
aMAP	0.5788	0.13	0.32–0.84	—
Alcohol Cohort				
5 y				
GALAD	0.8387	0.14	0.56–0.99	0.0003
ALBI	0.3474	0.13	0.09–0.61	—
aMAP	0.6715	0.17	0.33–0.99	—
7 y				
GALAD	0.7633	0.13	0.51–0.99	0.008
ALBI	0.4079	0.11	0.18–0.63	—
aMAP	0.6575	0.12	0.41–0.90	—
10 y				
GALAD	0.7363	0.11	0.51–0.96	0.002
ALBI	0.3956	0.10	0.20–0.59	—
aMAP	0.6707	0.11	0.46–0.88	—

^a^ Test of equality of ROC areas.

On competing risk analysis using other etiologies as the competing event, GALAD was very close to being a significant predictor at 10 years [SHR = 1.06, *p* = 0.006, 1.02–1.112 (95% C.I.)]. While with mortality as a competing event, curves are shown in Supplemental Figure 1, http://links.lww.com/HC9/A511.

## DISCUSSION

Improvement of the current strategy of surveillance programs in patients with cirrhosis is critical to increase patients’ survival. EASL guidelines currently recommend HCC surveillance for patients with cirrhosis with a 1.5% annual risk of HCC or higher. Because of the unsatisfactory sensitivity and specificity of AFP, a more frequent determination of AFP combined with more sophisticated imaging techniques, CT and MRI, is unlikely to be effective.^8,13^ Although there are concerns about the thresholds and how to estimate patients’ HCC risk, HCC risk-based surveillance seems to be the potential ideal, tailored approach to decreasing HCC deaths and costs, particularly when strategies combining the simultaneous use of multiple blood biomarkers are applied.

Multiple CHB-specific scores have been developed in Asian cohorts to stratify their risk of developing HCC during surveillance.^15^ In contrast, there have been few studies on predictive models of HCC risk scores in CHC patients, the main limitation being a lack of validation cohorts in the majority of them.^16^ The development of new biomarkers cannot be postponed due to the increase in costs as a result of false positive AFP elevations necessitating additional imaging investigations, posing a threat to the sustainability of surveillance programs.^8^ To meet this demand, various score systems (ALBI, aMAP, and more recently GALAD) have been proposed in recent decades, primarily in case-control studies, while only a few studies have validated their diagnostic accuracy in prospective studies.

In this study, we investigated the ability of 3 score systems to detect early HCC in a 12-year prospective study of patients with compensated advanced chronic liver disease. For the first time, our findings demonstrate that the GALAD score outperforms aMAP and ALBI in terms of diagnostic performance. This conclusion is supported by the 5, 7, and 10-year univariate analyses, although as expected, GALAD’s performance is lower at 10 years due to the long-time of observation.

GALAD is a combination of 3 different biomarkers, AFP, AFP-L3, and DCP, as well as age and gender, which has previously been shown to have high sensitivity for early HCC detection in patients with cirrhosis.^13^ Only 1 Italian study previously measured AFP, AFP-L3, and DCP levels in a small cohort of patients (44 CLD with no HCC and 54 HCC patients with HCV or HBV etiology). Serum biomarker levels were significantly higher in HCC patients than in CLD, with AUROCs of 88%, 87%, and 87%, respectively. Furthermore, the combination of AFP, AFP-L3, and DCP outperformed a single biomarker in detecting HCC,^17^ and also the competing risk analysis that had mostly confirmed the findings of Cox regression analysis, as GALAD outperformed the detection of HCC.

In a multicenter case-control study, the GALAD score’s ability to diagnose HCC was confirmed even in NASH patients, with a significantly higher AUROC than serum AFP, AFP-L3, or DCP alone (96% vs. 88%, 86%, and 87%, respectively). The AUROC values for the GALAD score were consistent both in patients with cirrhosis and without (93% and 98%, respectively).^18^ In our study, the performance of GALAD is superior to that of each individual biomarker, either due to the heterogeneity of HCC or the multifactorial etiologies underlying the tumor onset.^5,13^ This is of growing interest since the reduction in HCC occurrence over time in various nations due to the efficacy of antiviral therapeutic strategies and HBV vaccination programs may be offset or even surpassed in the future by the currently rising rates of metabolic syndrome, NASH, and alcohol abuse. For these, no score system has been demonstrated to be effective, emphasizing the need for additional HCC surveillance algorithms.^15,19^ Herein, we demonstrate that the GALAD score performed better in the alcohol group throughout the follow-up period. This may be a milestone for the future management of these patients. On the contrary, in the NASH cohort, GALAD did not perform significantly better than the other scores, although a trend was observed. However, this might be due to the particularly high heterogeneity of potential molecular mechanisms involved in carcinogenesis, suggesting that very large numbers of patients with this etiology need to be investigated in further studies.

A future direction combining more biomarkers, independently of type and origin, may be the most appropriate strategy in oncology, including HCC, showing that we still lack a score system accepted as the standard of care.^15^ Indeed, in our study, combining the GALAD score with the routinely used tests (albumin, bilirubin, and platelets) included in the ALBI and aMAP score did not improve the prediction of HCC development in any of the sub-cohort analyzed, further underlining the power of the pure GALAD system.

In conclusion, the number of Europeans dying of HCC is rising, with 78,400 being predicted in the next few years (23,000 in Central-Eastern Europe, 10,500 in Northern Europe, 21,200 in Southern Europe, and 23,700 in Western Europe), reaching a total of 78,400 deaths, owing to the shift from viral to nonviral causes. HCC surveillance of patients with cirrhosis aims to reduce this excess mortality (https://easl.eu/publication/easl-policy-statement-risk-based/). The prediction of HCC development would play a crucial role in reducing mortality, and from this perspective, GALAD is currently the most appropriate score for use in surveillance strategies.

## Supplementary Material
