# Supplementary material for: GALAD outperforms aMAP and ALBI for predicting HCC in patients with compensated advanced chronic liver disease: A 12-year prospective study
Source: Hepatol Commun. 2023 Sep 15;7(10):e0262. doi: 10.1097/HC9.0000000000000262 (PMC10503687; doi:10.1097/HC9.0000000000000262)
Supplement: Supplementary file 1 [file hc9-7-e0262-s001.docx]

**Manuscript ID HEP4-23-0603**

Supplementary Table 1. Cox regression model for HCC prediction at 5, 7 and 10 years on single parameters, in viral cohort.

| Parameters | 5 years | | | | 7 years | | | | 10 years | | | |
| --- | --- | --- | --- | --- | --- | --- | --- | --- | --- | --- | --- | --- |
|  | HR | p-value | 95% (C.I.) | C-Index | HR | p-value | 95% (C.I.) | C-Index | HR | p-value | 95% (C.I.) | C-Index |
| Age | 1.02 | 0.29 | 0.98 to 1.06 | 0.51 | 1.01 | 0.41 | 0.41 to 1.04 | 0.51 | 1.01 | 0.28 | 0.99 to 1.04 | 0.52 |
| Gender (M) | 1.85 | 0.21 | 0.70 to 4.89 | 0.55 | 1.79 | 0.14 | 0.83 to 3.88 | 0.57 | 1.74 | 0.12 | 0.87 to 3.47 | 0.56 |
| BMI | 1.05 | 0.38 | 0.94 to 1.16 | 0.60 | 1.03 | 0.41 | 0.95 to 1.13 | 0.53 | 1.06 | 0.09 | 0.99 to 1.14 | 0.55 |
| Diabetes | 0.89 | 0.84 | 0.31 to 2.60 | 0.48 | 0.99 | 0.99 | 0.44 to 2.25 | 0.49 | 0.93 | 0.86 | 0.44 o 1.99 | 0.49 |
| Viral Etiology | -- | -- | -- | -- | -- | -- | -- | -- | -- | -- | -- | -- |
| Albumin | 0.37 | **0.008** | 0.18 to 0.77 | 0.62 | 0.44 | **0.006** | 0.24 to 0.79 | 0.60 | 0.48 | **0.008** | 0.28 to 0.82 | 0.59 |
| Bilirubin | 1.02 | 0.92 | 0.73 to 1.41 | 0.55 | 1.01 | 0.91 | 0.79 to 1.30 | 0.54 | 1.01 | 0.94 | 0.80 to 1.27 | 0.54 |
| AST | 0.99 | 0.13 | 0.98 to 1.00 | 0.58 | 0.99 | 0.80 | 0.99 to 1.01 | 0.48 | 0.99 | 0.50 | 0.99 to 1.00 | 0.49 |
| ALT | 0.99 | 0.34 | 0.99 to 1.00 | 0.48 | 0.99 | 0.73 | 0.99 to 1.00 | 0.51 | 0.99 | 0.31 | 0.99 to 1.00 | 0.55 |
| AFP | 1.00 | 0.06 | 0.99 to 1.01 | 0.61 | 1.00 | 0.10 | 0.99 to 1.01 | 0.55 | 1.00 | **<0.001** | 1.00 to 1.01 | 0.54 |
| AFP-L3 | 1.02 | 0.41 | 0.98 to 1.05 | 0.59 | 1.01 | 0.46 | 0.98 to 1.05 | 0.55 | 1.00 | 0.77 | 0.97 to 1.04 | 0.51 |
| DCP | 0.99 | 0.64 | 0.97 to 1.02 | 0.29 | 0.99 | 0.52 | 0.97 to 1.02 | 0.34 | 0.99 | 0.59 | 0.98 to 1.01 | 0.37 |
| *Scores* |  |  |  |  |  |  |  |  |  |  |  |  |
| MELD | 1.02 | 0.69 | 0.91 to 1.15 | 0.54 | 1.00 | 0.94 | 0.91 to 1.11 | 0.52 | 0.99 | 0.99 | 0.91 to 1.09 | 0.48 |
| Child-Pugh | 1.64 | 0.25 | 0.70 to 3.82 | 0.53 | 1.93 | **0.03** | 1.07 to 3.51 | 0.57 | 1.71 | 0.06 | 0.97 to 3.02 | 0.56 |
| ALBI Score | 2.20 | **0.02** | 1.13 to 4.30 | 0.61 | 1.90 | **0.02** | 1.11 to 3.26 | 0.58 | 1.80 | **0.02** | 1.10 to 2.95 | 0.58 |
| aMAP | 1.11 | **0.01** | 1.02 to 1.20 | 0.57 | 1.04 | 0.21 | 0.98 to 1.10 | 0.50 | 1.05 | 0.08 | 0.99 to 1.11 | 0.53 |
| GALAD | 1.33 | **0.008** | 1.08 to 1.64 | 0.67 | 1.27 | **0.004** | 1.08 to 1.49 | 0.63 | 1.24 | **0.004** | 1.07 to 1.44 | 0.64 |
| GALAD/Alb/Bil (z-score) | 1.13 | 0.24 | 0.92 to 1.39 | 0.60 | 1.08 | 0.37 | 0.91 to 1.26 | 0.54 | 1.07 | 0.33 | 0.93 to 1.24 | 0.56 |
| GALAD/Alb/Bil/Plt (z-score) | 1.09 | 0.37 | 0.90 to 1.31 | 0.59 | 1.08 | 0.28 | 0.94 to 1.25 | 0.57 | 1.07 | 0.30 | 0.94 to 1.22 | 0.57 |

Abbreviations: HR, Hazard Ratio; 95% (C.I.), Confidential Interval at 95%; C-Index, Harrell C-index.

Supplementary Table 2. Multiple Cox regression model for HCC prediction at 5, 7, and 10 years, in viral cohort.

| Parameters | 5 years | | | | 7 years | | | | 10 years | | | |
| --- | --- | --- | --- | --- | --- | --- | --- | --- | --- | --- | --- | --- |
|  | HR | p-value | 95% (C.I.) | C-Index | HR | p-value | 95% (C.I.) | C-Index | HR | p-value | 95% (C.I.) | C-Index |
| Albumin | -- | -- | -- | -- | -- | -- | -- | -- | 0.44 | 0.006 | 0.24 to 0.79 | 0.63 |
| AFP | -- | -- | -- |  | -- | -- | -- |  | 1.00 | <0.001 | 1.00 to 1.01 |  |
| *Scores* |  |  |  |  |  |  |  |  |  |  |  |  |
| Child-Pugh | -- | -- | -- |  | 1.19 | 0.72 | 0.46 to 3.08 | 0.62 | -- | -- | -- | 0.62 |
| ALBI Score | 2.15 | 0.09 | 0.89 to 5.17 | 0.66 | 1.87 | 0.11 | 0.86 to 4.05 |  | 1.94 | 0.02 | 1.12 to 3.56 |  |
| aMAP | 1.04 | 0.40 | 0.95 to 1.14 |  | -- | -- | -- |  | -- | -- | -- |  |
| GALAD | 1.27 | 0.04 | 1.01 to 1.60 |  | 1.20 | 0.03 | 1.02 to 1.42 |  | 1.19 | 0.02 | 1.02 to 1.39 |  |

Abbreviations: HR, Hazard Ratio; 95% (C.I.), Confidential Interval at 95%; C-Index, Harrell C-index.

Supplementary Table 3. Cox regression model for HCC prediction at 5, 7 and 10 years on single parameters, in NASH cohort.

| Parameters | 5 years | | | | 7 years | | | | 10 years | | | |
| --- | --- | --- | --- | --- | --- | --- | --- | --- | --- | --- | --- | --- |
|  | HR | p-value | 95% (C.I.) | C-Index | HR | p-value | 95% (C.I.) | C-Index | HR | p-value | 95% (C.I.) | C-Index |
| Age | 1.06 | 0.26 | 0.95 to 1.18 | 0.60 | 1.06 | 0.13 | 0.98 to 1.15 | 0.64 | 1.05 | 0.18 | 0.98 to 1.13 | 0.62 |
| Gender (M) | 0.94 | 0.94 | 0.18 to 4.90 | 0.56 | 0.80 | 0.72 | 0.23 to 2.78 | 0.57 | 0.81 | 0.71 | 0.27 to 2.45 | 0.58 |
| BMI | 0.91 | 0.39 | 0.73 to 1.13 | 0.49 | 1.02 | 0.75 | 0.88 to 1.19 | 0.74 | 0.97 | 0.61 | 0.86 to 1.09 | 0.33 |
| Diabetes | 2.29 | 0.32 | 0.44 to 11.94 | 0.65 | 2.42 | 0.19 | 0.64 to 9.16 | 0.68 | 2.19 | 0.19 | 0.68 to 7.02 | 0.67 |
| Viral Etiology | 2.27 | 0.47 | 0.24 to 21.07 | 0.49 | 1.85 | 0.57 | 0.21 to 16.04 | 0.49 | 1.32 | 0.79 | 0.16 to 10.75 | 0.49 |
| Albumin | 0.38 | 0.23 | 0.08 to 1.84 | 0.71 | 0.31 | **0.05** | 0.09 to 1.01 | 0.79 | 0.49 | 0.17 | 0.18 to 1.35 | 0.73 |
| Bilirubin | 1.95 | 0.18 | 0.74 to 5.12 | 0.69 | 1.32 | 0.39 | 0.70 to 2.46 | 0.69 | 1.14 | 0.70 | 0.59 to 2.20 | 0.64 |
| AST | 0.90 | 0.10 | 0.80 to 1.02 | 0.74 | 0.99 | 0.71 | 0.96 to 1.03 | 0.42 | 0.99 | 0.94 | 0.98 to 1.02 | 0.42 |
| ALT | 0.97 | 0.25 | 0.93 to 1.02 | 0.65 | 0.98 | 0.18 | 0.94 to 1.01 | 0.55 | 0.99 | 0.33 | 0.96 to 1.01 | 0.57 |
| AFP | 0.99 | 0.90 | 0.92 to 1.08 | 0.57 | 1.00 | 0.89 | 0.97 to 1.03 | 0.61 | 1.00 | 0.75 | 0.98 to 1.03 | 0.67 |
| AFP-L3 | 1.48 | 0.14 | 0.88 to 2.49 | 0.77 | 1.07 | 0.50 | 0.88 to 1.30 | 0.41 | 1.10 | 0.25 | 0.93 to 1.29 | 0.46 |
| DCP | 0.97 | 0.86 | 0.72 to 1.32 | 0.49 | 0.99 | 0.86 | 0.98 to 1.01 | 0.29 | 0.99 | 0.86 | 0.98 to 1.01 | 0.28 |
| *Scores* |  |  |  |  |  |  |  |  |  |  |  |  |
| MELD | 1.17 | 0.21 | 0.91 to 1.49 | 0.67 | 1.09 | 0.34 | 0.91 to 1.30 | 0.69 | 1.07 | 0.44 | 0.90 to 1.28 | 0.66 |
| Child-Pugh | 1.05 | 0.96 | 0.15 to 7.22 | 0.45 | 2.02 | 0.12 | 0.84 to 4.86 | 0.71 | 1.59 | 0.28 | 0.68 to 3.71 | 0.67 |
| ALBI Score | 2.71 | 0.17 | 0.66 to 11.21 | 0.71 | 2.88 | **0.05** | 0.99 to 8.33 | 0.78 | 1.90 | 0.18 | 0.75 to 4.80 | 0.72 |
| aMAP | 1.13 | 0.11 | 0.97 to 1.31 | 0.61 | 1.10 | 0.10 | 0.98 to 1.22 | 0.66 | 1.08 | 0.11 | 0.98 to 1.18 | 0.63 |
| GALAD | 1.07 | 0.80 | 0.65 to 1.75 | 0.30 | 1.21 | 0.22 | 0.89 to 1.66 | 0.65 | 1.24 | 0.13 | 0.94 to 1.65 | 0.66 |
| GALAD/Alb/Bil (z-score) | 1.70 | 0.33 | 0.58 to 4.98 | 0.57 | 1.47 | 0.06 | 0.98 to 2.20 | 0.87 | 1.46 | **0.04** | 1.01 to 2.11 | 0.81 |
| GALAD/Alb/Bil/Plt (z-score) | 1.21 | 0.66 | 0.52 to 2.79 | 0.52 | 1.41 | 0.14 | 0.89 to 2.23 | 0.84 | 1.35 | 0.14 | 0.90 to 2.03 | 0.75 |

Abbreviations: HR, Hazard Ratio; 95% (C.I.), Confidential Interval at 95%; C-Index, Harrell C-index.

Supplementary Table 4. Cox regression model for HCC prediction at 5, 7 and 10 years on single parameters, in Alcohol cohort.

| Parameters | 5 years | | | | 7 years | | | | 10 years | | | |
| --- | --- | --- | --- | --- | --- | --- | --- | --- | --- | --- | --- | --- |
|  | HR | p-value | 95% (C.I.) | C-Index | HR | p-value | 95% (C.I.) | C-Index | HR | p-value | 95% (C.I.) | C-Index |
| Age | 1.28 | **0.02** | 1.04 to 1.57 | 0.91 | 1.16 | **0.01** | 1.03 to 1.29 | 0.75 | 1.13 | **0.009** | 1.03 to 1.23 | 0.74 |
| Gender (M) | -- | -- | -- | -- | 1.11 | 0.92 | 0.13 to 9.61 | 0.54 | 1.57 | 0.67 | 0.19 to 12.86 | 0.55 |
| BMI | 1.02 | 0.86 | 0.83 to 1.25 | 0.29 | 0.89 | 0.33 | 0.71 to 1.12 | 0.78 | 0.96 | 0.67 | 0.82 to 1.13 | 0.66 |
| Diabetes | 1.29 | 0.83 | 0.12 to 14.31 | 0.48 | 1.28 | 0.77 | 0.23 to 7.01 | 0.55 | 0.85 | 0.85 | 0.17 to 4.24 | 0.49 |
| Viral Etiology | -- | -- | -- | -- | -- | -- | -- | -- | -- | -- | -- | -- |
| Albumin | 1.14 | 0.90 | 0.14 to 8.92 | 0.59 | 1.13 | 0.87 | 0.24 to 5.32 | 0.50 | 0.74 | 0.58 | 0.25 to 2.16 | 0.57 |
| Bilirubin | 0.86 | 0.72 | 0.39 to 1.92 | 0.46 | 1.08 | 0.27 | 0.94 to 1.24 | 0.69 | 1.06 | 0.45 | 0.90 to 1.25 | 0.58 |
| AST | 1.03 | 0.20 | 0.98 to 1.07 | 0.79 | 1.04 | **0.01** | 1.01 to 1.07 | 0.77 | 1.03 | **0.02** | 1.00 to 1.05 | 0.73 |
| ALT | 1.05 | **0.01** | 1.01 to 1.09 | 0.73 | 1.03 | **0.01** | 1.01 to 1.06 | 0.68 | 1.02 | 0.09 | 0.99 to 1.05 | 0.53 |
| AFP | 1.06 | 0.49 | 0.90 to 1.24 | 0.63 | 1.03 | 0.72 | 0.88 to 1.19 | 0.62 | 1.01 | 0.85 | 0.88 to 1.17 | 0.61 |
| AFP-L3 | 1.05 | 0.57 | 0.88 to 1.25 | 0.54 | 0.97 | 0.83 | 0.75 to 1.26 | 0.55 | 1.02 | 0.84 | 0.84 to 1.23 | 0.49 |
| DCP | -- | -- | -- | -- | -- | -- | -- | -- | -- | -- | -- | -- |
| *Scores* |  |  |  |  |  |  |  |  |  |  |  |  |
| MELD | 1.05 | 0.59 | 0.88 to 1.26 | 0.62 | 1.09 | 0.17 | 0.96 to 1.24 | 0.72 | 1.06 | 0.34 | 0.94 to 1.19 | 0.66 |
| Child-Pugh | 1.01 | 0.99 | 0.14 to 7.10 | 0.46 | 1.98 | 0.28 | 0.57 to 6.85 | 0.59 | 1.87 | 0.24 | 0.66 to 5.35 | 0.60 |
| ALBI Score | 0.92 | 0.93 | 0.14 to 6.20 | 0.57 | 1.49 | 0.53 | 0.42 to 5.23 | 0.56 | 1.54 | 0.41 | 0.54 to 4.38 | 0.59 |
| ALBI Score inv | 1.57 | 0.84 | 0.02 to 124.23 | 0.57 | 0.95 | 0.95 | 0.19 to 4.82 | 0.57 | 0.91 | 0.89 | 0.22 to 3.78 | 0.59 |
| aMAP | 1.12 | 0.23 | 0.93 to 1.36 | 0.58 | 1.14 | 0.08 | 0.99 to 1.31 | 0.68 | 1.14 | **0.03** | 1.01 to 1.28 | 0.69 |
| GALAD | 7.43 | **0.01** | 1.63 to 33.86 | 0.99 | 3.85 | **0.001** | 1.77 to 8.36 | 0.89 | 3.12 | **0.001** | 1.63 to 5.99 | 0.87 |
| GALAD/Alb/Bil (z-score) | 4.97 | **0.02** | 1.31 to 18.86 | 0.96 | 4.91 | **0.001** | 1.97 to 12.26 | 0.97 | 3.53 | **<0.001** | 1.76 to 7.07 | 0.91 |
| GALAD/Alb/Bil/Plt (z-score) | 3.38 | **0.01** | 1.34 to 8.54 | 0.92 | 2.82 | **0.001** | 1.57 to 5.07 | 0.92 | 2.26 | **0.001** | 1.40 to 3.66 | 0.85 |

Abbreviations: HR, Hazard Ratio; 95% (C.I.), Confidential Interval at 95%; C-Index, Harrell C-index.

Supplementary Table 5. Multiple Cox regression model for HCC prediction at 5, 7, and 10 years, in alcohol cohort.

| Parameters | 5 years | | | | 7 years | | | | 10 years | | | |
| --- | --- | --- | --- | --- | --- | --- | --- | --- | --- | --- | --- | --- |
|  | HR | p-value | 95% (C.I.) | C-Index | HR | p-value | 95% (C.I.) | C-Index | HR | p-value | 95% (C.I.) | C-Index |
| Age | 1.62 | 0.10 | 0.91 to 2.89 | 0.98 | 1.20 | 0.01 | 1.04 to 1.39 | 0.85 | 1.16 | 0.004 | 1.05 to 1.29 | 0.81 |
| AST | -- | -- | -- |  | 1.04 | 0.06 | 0.99 to 1.08 |  | 1.047 | 0.002 | 1.01 to 1.07 |  |
| ALT | 1.15 | 0.14 | 0.96 to 1.39 |  | 1.03 | 0.21 | 0.98 to 1.08 |  | -- | -- | -- |  |
| *Scores* |  |  |  |  |  |  |  |  |  |  |  |  |
| aMAP | -- | -- | -- | -- | -- | -- | -- | -- | 1.04 | 0.65 | 0.88 to 1.22 | 0.87 |
| GALAD | -- | -- | -- |  | -- | -- | -- |  | 2.79 | 0.008 | 1.31 to 5.93 |  |

Abbreviations: HR, Hazard Ratio; 95% (C.I.), Confidential Interval at 95%; C-Index, Harrell C-index.


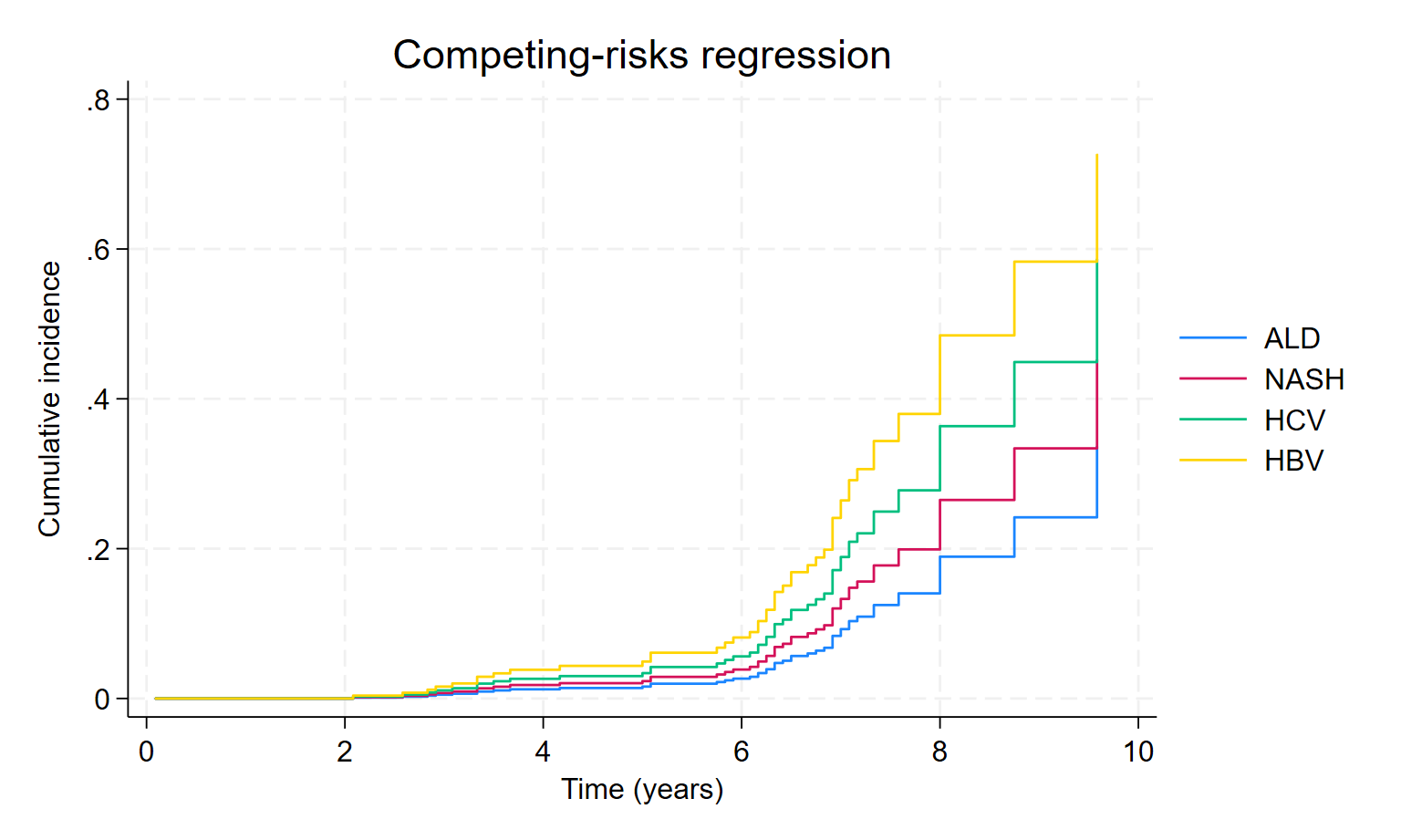


Supplementary Figure 1. Cumulative incidence with mortality as competing risk, stratified for etiology.
